# EHMTI-0307. Chronification of migraine: a clinical and voxel-based morphometry study

**DOI:** 10.1186/1129-2377-15-S1-E43

**Published:** 2014-09-18

**Authors:** S Yu, X Chen, Z Chen, ZHAO Dong, LIN Ma

**Affiliations:** 1 Department of Neurology, Chinese PLA General Hospital, Beijing, China; 2 Department of Radiology, Chinese PLA General Hospital, Beijing, China

## Introduction

Migraine is an episodic disease which may transform to a chronic form. However, the precise pathogenesis of migraine chronification is not well understood.

## Aim

We aimed to detect the changes of brain gray matter volume (GMV) in chronification of migraine.

## Methods

Voxel-based morphometry (VBM) of MRI was employed to analyze the volume of brain gray matter in 44 patients with chronic migraine with medication-overuse headache (CM-MOH), 16 patients with CM without MOH (CMwoMOH), 18 patients with episodic migraine (EM) and 32 healthy controls (HCs).

## Results

The GMV did not differ significantly among groups but was positively related to body mass index (BMI) and male gender, negatively related to age, course of disease and anxiety. Compared with HCs, local GMV of middle temporal pole decreased in all the three patient groups. GMV of right superior and middle orbitofrontal gyrus and right inferior temporal gyrus decreased in CM-MOH and CMwoMOH groups. Besides, GMV of left gyrus rectus, middle cingulate gyrus, bilateral insula, right Rolandic Operum, occipital lobes decreased in CM-MOH group. GMV of right middle frontal gyrus, Rolandic Operum, right precentral gyrus, left postcentral gyrus, left superior occipital lobe increased in patients with EM and CMwoMOH. Furthermore, CMwoMOH patients had significantly increased GMV in bilateral caudate nucleus.

## Conclusion

Our findings indicate that a reduced volume of orbitofrontal gyrus may reflect chronicty of migraine. Decreased GMV of gyrus rectus may reflect disease-specific modifications of CM-MOH. Increased GMV of caudate nucleus may specifically reflect pathophysiological changes of chronicity of migraine without medication overuse.

No conflict of interest.

